# Guest Edited Collection: Gravitational biology and space medicine

**DOI:** 10.1038/s41598-019-51231-8

**Published:** 2019-10-08

**Authors:** Daniela Grimm

**Affiliations:** 1 Clinic and Policlinic for Plastic, Aesthetic and Hand Surgery, Otto von Guericke University Magdeburg, 39120 Magdeburg, Germany; 2 Department for Biomedicine, Aarhus University, Høegh-Guldbergsgade 10, DK-8000 Aarhus C, Denmark; 3 Gravitational Biology and Translational Regenerative Medicine, Faculty of Medicine and Mechanical Engineering, Otto von Guericke University Magdeburg, 39120 Magdeburg, Germany

**Keywords:** Extracellular signalling molecules, Molecular medicine

## Abstract

A return to the Moon, Mars expeditions, and a rise in space tourism will lead to an increasing number of human spaceflights. The *‘Gravitational biology and space medicine’* Collection focuses on the challenges to the health of humans in space during long-term space missions and the physiological changes during short-term altered gravity conditions, the possible influence of space radiation, available countermeasures and possible applications on Earth. In addition, studies reporting on *in vivo* changes in space-flown mice were published. Finally, this Collection also brings together articles reporting experiments using cells cultured under conditions of real microgravity on the International Space Station, or exposed in ground-based facilities, in order to study morphological and molecular alterations in different cell types.

Experiencing a spaceflight is a dream of many enthusiasts and those working in space research. However, while a space journey is fascinating, unfortunately, it can cause various short- and long-term health concerns. Human and animal bodies undergo dramatic changes after an extended stay on the International Space Station (ISS)^1,2^.

Space medicine deals with the physiological and biological effects of spaceflight on the human organism, its organs, tissues and cells^1,3–5^. It includes administering medicine to astronauts, cosmonauts, taikonauts and private space travellers in outer space and on Earth, along with the development of countermeasures for the various health problems of humans in space.

For medical and biotechnological reasons, it is important to study animals and mammalian cells that are exposed to real microgravity (r-µ*g*) in space. It is necessary to detect the cellular changes responsible for the various medical problems often observed in humans on the ISS. The cellular response to microgravity (µ*g*) has been partially described for humans and animal models, whereas the intracellular regulatory mechanisms are still a mystery. Studies on whole organisms, cells and tissues are not only complementary but essential for a holistic interpretation. In such models, several molecular mechanisms involved in biological processes have been characterised^3–6^.

Studies of tissues and cells exposed to r-µ*g* are rare and rather costly. For this reason, researchers have developed simulation devices to generate µ*g* on Earth, such as the NASA-developed rotating wall vessel (RWV) bioreactor, the random positioning machine (RPM), the magnetic levitator, and the two- (2D) and three-dimensional (3D) clinostats, to prepare for spaceflights and to perform ground-based µ*g* experiments on stem and other specialised cells^7–10^. In addition, the National Aeronautics and Space Administration (NASA), the European Space Agency (ESA) and other agencies offer the possibility of short-term µ*g* experiences, such as parabolic flight (PF)^11–13^ and sounding rocket missions^12^.

The Guest Edited Collection ‘*Gravitational biology and space medicine*’ includes publications reporting on health concerns for astronauts and cosmonauts^14–16^ during space missions, and physiological changes caused by short-term µ*g*^17^. Several studies investigating the impact of µ*g* on the behaviour and health of mice on the ISS were published in this Collection^18–23^, in addition to investigations at the cellular level in space^24^ and in short-term µ*g* provided by PF maneuvers^25^. Moreover, cell studies in simulated (s-) µ*g* at ground-based facilities are included in this Collection^26–30^.

Humans in space can experience various health problems. Immunosuppression in spaceflight increases the risk of opportunistic infections. Crewmembers are exposed to different stressors, potentially altering the composition of their microbiomes. Voorhies *et al*.^14^ investigated nine astronauts, and showed that the composition of the astronauts’ microbiome (gastrointestinal tract, skin, nose and tongue) was changed during space travel. Notably, the altered skin microbiome might be involved in the frequently observed skin rashes and hypersensitivity episodes of astronauts. These findings warrant further detailed examination before deeper space exploration starts. Moore *et al*.^15^ demonstrated that long-duration spaceflight adversely affects post-landing operator proficiency. Eight crewmembers experienced general discomfort in motor function and motion perception after a mission, and a lack of cognitive reserve. This only manifested in duplicate tasks that recovered to baseline four days after landing^15^. These results suggest that the development of countermeasures should target the cumulative effect of the subtle physiological changes observed on landing day. Recommendations for countermeasures were published in Moore *et al*.^15^. Reynolds *et al*.^16^, investigated 301 astronauts and 117 cosmonauts, demonstrating that space radiation has no strong impact on their mortality. They showed that survival estimates from Kaplan-Meier curves were largely congruent with those of competing risk methods, suggesting that if ionising radiation impacts the risk of death due to cancer and cardiovascular disease, the effect is not dramatic^16^. However, the authors concluded that future deep space exploration might provide larger doses of space radiation, leading to a different risk profile for future astronauts and cosmonauts; therefore, it is important to continue their surveillance for potential harmful effects of such exposure^16^.

Investigations of the human stance during PF maneuvers compared to 1*g-*conditions revealed the similarity of postural recovery responses in 1*g*-, hypo- and hyper-gravity conditions^17^. These results showed that muscle synergies and segmental strategies acquired on Earth are robust and persistent across variable acute changes in gravitation^17^.

The NASA Rodent Habitat on the ISS was used to study the behaviour of mice in r-µ*g*, and for investigating changes in different tissues^18–23^. The detailed behavioural analysis of these mice provides a useful model system for increasing our knowledge of human responses to spaceflight^18^. These studies provide the opportunity to examine how physical movement influences responses to µ*g*^18^.

Ogneva *et al*.^19^ studied the vas deferens and testes tissues of space-flown mice. They found that during the 3-week spaceflight an adaptive protein profile formed in the germinative tissue of male mice^19^. In addition, gene expression of Tet2 demethylase was increased and histone deacetylase (Hdac1) decreased^19^. The mRNA expression changes might be important during the early period of readaptation, since they could lead to an increase in the expression of target genes^19^.

Spaceflight is associated with loss of muscle mass and strength^1^. Cadena *et al*.^20^ investigated E3 ubiquitin ligase MuRF1 (Muscle-specific RING finger 1) null KO mice in space on the ISS and by the hindlimb suspension model. The muscle of MuRF1 null mice was protected in the ground-based model. By contrast, skeletal muscle atrophy in these mice was enhanced by unique mechanisms in space^20^.

Another important health concern for crewmembers is spaceflight-induced structural and functional damage of the eyes. Mao *et al*. investigated mice on the ISS^21^. They demonstrated blood-retinal barrier (BRB) disruption and ocular adaptation after the 35-day mission. Post-flight proteomics studies revealed changes in cell death (apoptosis), cell cycle, immune responses, mitochondrial function and metabolic stress in space-flown mice compared to ground-based control animals^21^. The results of this study show a complex cellular response influencing the structure of the retina and BRB integrity after the 35-day space mission^21^. A second study assessing eye changes after spaceflight identified 600 differentially expressed genes by RNA sequencing in mouse retinas^22^. The differentially expressed genes were associated with retinitis pigmentosa and changes in chromatin structure. In addition, the thicknesses of the retina, the retinal pigment epithelium and the choroid layer were lower after spaceflight, indicating that retinal changes cause the visual problems^22^. Finally, whole-transcript cDNA sequencing showed that a 35-day stay in orbit on ISS may alter the homeostatic gene expression of mice spleens by a combination of µ*g* and other environmental stressors^23^.

The Collection also covers cell and molecular biological studies in space, and for short-term µ*g* episodes provided by PFs. Gambacurta *et al*.^24^ showed that rapamycin induces transcriptional activation of human blood-derived stem cells (BDSCs) towards osteogenic differentiation in space. These findings, applying proteomics and epigenetic methods, may represent the first step in identifying new biomarkers and therapeutic targets to treat osteoporosis in space and on Earth^24^.

A PF study examined the attachment of peripheral blood mononuclear cells to adhesion molecules under flow conditions, and antigen-induced immune activation in whole blood^25^. The flow and rolling speed of cells were moderately accelerated during µ*g* periods, which were accompanied by a clear reduction in rolling rate. After the PF, whole blood tests indicated monocytes were in a “primed” state, with potentiated antigen-induced pro-inflammatory cytokine responses. In addition, anti-inflammatory cytokines were elevated, and monocytes showed a surface molecule pattern indicating immunosuppression. The results suggest an immunologic counterbalance to avoid disproportionate immune responses.

3D clinorotation was used to study mesenchymal stromal cell (MSC) response to inflammatory stimulation^26^. A reduced response of MSCs to priming in µ*g* might also indicate less involvement of these cells in tissue remodelling^26^. Arun *et al*.^27^ studied the effects of s-µ*g* on increasing stemness in human colorectal cancer cells (HCT116), using a rotary cell culture system (RCCS). This study highlighted the importance of µ*g* and physical factors in the regulation of stemness in colorectal cancer cells^27^. Cancer research in space is currently a popular area of study^29^, with the prospect of researchers developing new cancer treatment strategies. Notably, Costantini *et al*.^28^ showed that RCCS-exposure promoted cells to form 3D spheroids. It also stimulated pluripotency and a glycolytic metabolism in human hepatic and biliary tree stem/progenitor cells^28^. These results are important for stem cell biology, and confirm earlier results where other cell types were exposed to µ*g*^6,7^. Cho *et al*.^30^ examined endometrial stroma cells exposed to 3D clinorotation and showed that µ*g* inhibited decidualisation by reducing forkhead box O3 (FOXO3a) expression, autophagic flux and proliferation, as well as decreasing protein kinase B (Akt) activity, matrix metallopeptidase 2 (MMP2) expression and migration. They suggested that µ*g* during spaceflight might lead to an unreceptive endometrium by suppressing decidualisation potential^30^.

Finally, an animal study investigated the early effects of ^56^Fe irradiation in mice, showing specific short-term changes in neuropathology and behaviour^31^, with differences in dose, sex, and disease predilection. Therefore, future studies assessing the risks of space radiation exposure seem to be important for space travel moving forward.

In conclusion, the ‘*Gravitational biology and space medicine*’ Collection gives a broad overview of the different space research topics (Fig. 1). It comprises *in vivo* and *in vitro* studies investigating health problems for crewmembers, but also includes basic scientific cell experiments and studies with *Euglena gracilis* exposed to PF maneuvers, where hierarchical clustering showed that changes were induced by the different accelerations^32^.

**Figure 1:**
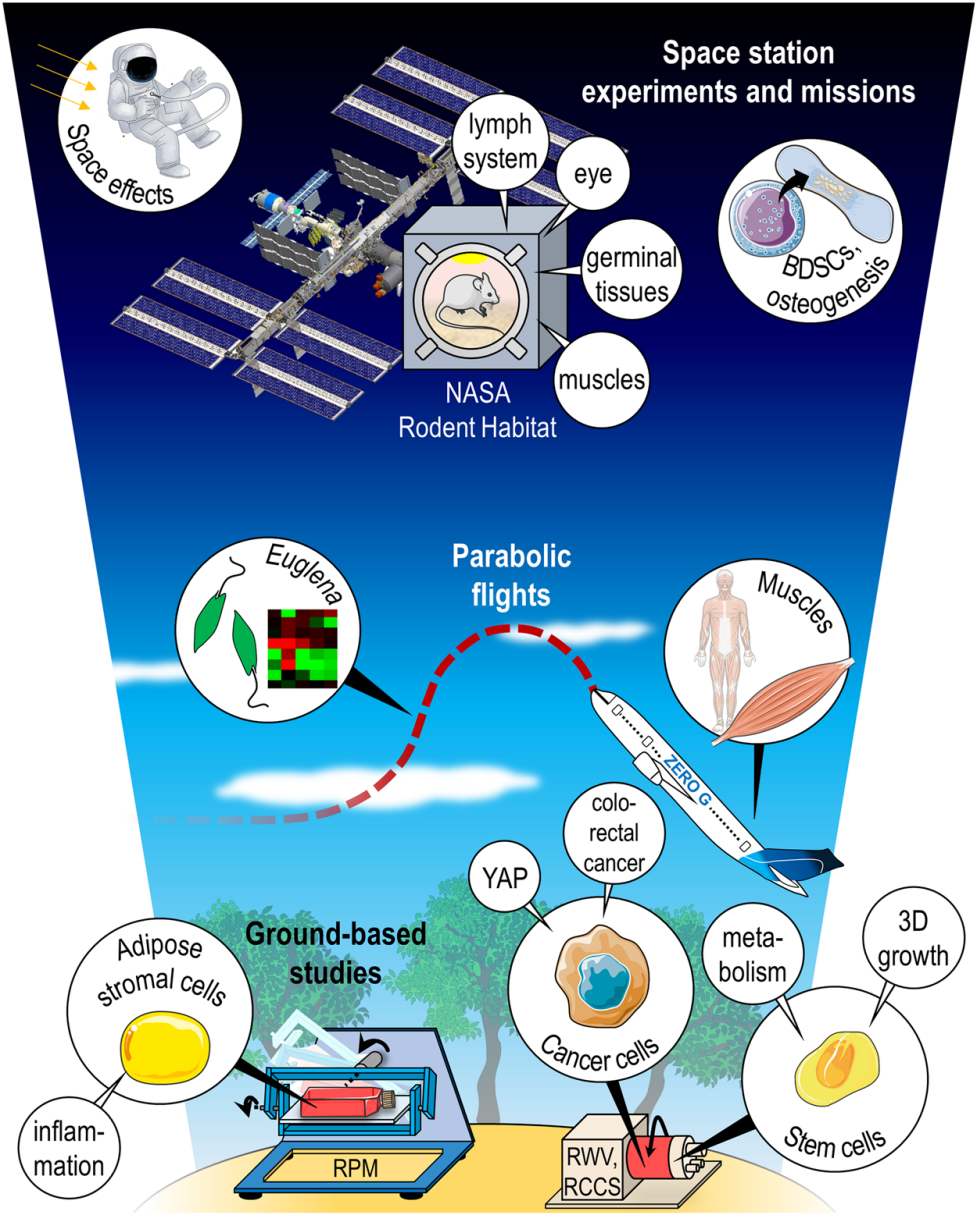
Compilation of studies contributed to the Collection ‘*Gravitational biology and space medicine*’. Parts of the figure were drawn by using pictures from Servier Medical Art (http://smart.servier.com/), licensed under a Creative Commons Attribution 3.0 Unported License (https://creativecommons.org/licenses/by/3.0/).
