# Publisher Correction: Guest Edited Collection: Gravitational biology and space medicine

**DOI:** 10.1038/s41598-019-54135-9

**Published:** 2019-11-19

**Authors:** Daniela Grimm

**Affiliations:** 1 Clinic and Policlinic for Plastic, Aesthetic and Hand Surgery, Otto von Guericke University Magdeburg, 39120 Magdeburg, Germany; 2 Department for Biomedicine, Aarhus University, Høegh-Guldbergsgade 10, DK-8000 Aarhus C, Denmark; 3 Gravitational Biology and Translational Regenerative Medicine, Faculty of Medicine and Mechanical Engineering, Otto von Guericke University Magdeburg, 39120 Magdeburg, Germany

Correction to: *Scientific Reports* 10.1038/s41598-019-51231-8, published online 08 October 2019

In this Editorial, the Author Contribution, Competing Interests and Acknowledgments sections were omitted.

The Author Contributions section should read:

“D.G. wrote the invited editorial.”

The Competing Interests section should read:

“The author declares no competing financial or non-financial interests.”

The Acknowledgements section should read:

“The author was supported by the German Space Agency (DLR, grants 50WB1524 and 50WB1924) and Aarhus University. I would like to thank Dr. Marcus Krüger for preparing Figure 1 and for his help with Endnote. Moreover, I would like to thank the team of PRS & EJE (Letchworth Garden City, UK) for academic proofreading of the manuscript.”

